# Prevalence and Correlates of Alcohol Use among a Sample of Nigerian Semirural Community Dwellers in Nigeria

**DOI:** 10.1155/2016/2831594

**Published:** 2016-04-19

**Authors:** Victor Olufolahan Lasebikan, Bolanle Adeyemi Ola

**Affiliations:** ^1^Department of Psychiatry, College of Medicine, University of Ibadan, PMB 5116, Ibadan, Nigeria; ^2^Department of Behavioural Sciences, Lagos State University Teaching Hospital, P.O. Box 0001, Lagos, Nigeria

## Abstract

*Objective*. To determine the prevalence and correlates of alcohol use among a sample of Nigerian semirural community dwellers in Nigeria.* Methods*. In a single arm nonrandomized intervention study, the assessment of baseline hazardous and harmful alcohol use and associated risk factors was conducted in two semirural local government areas of Oyo State, Nigeria, with the Alcohol, Smoking and Substance Involvement Screening Test (ASSIST). Participants included 1203 subjects 15 years and older, recruited between October 2010 and April 2011. ASSIST score of 0–10 was classified as lower risk scores, 11–26 as moderate risk, and 27+ as high risk.* Results*. Prevalence of lifetime alcohol use was 57.9% and current alcohol use was 23.7%. Current alcohol use was more prevalent among the younger age group *p* = 0.02, male gender *p* = 0.003, unmarried *p* < 0.01, low educational level *p* = 0.003, low socioeconomic class *p* = 0.01, unemployed *p* < 0.001, and the Christians *p* < 0.01. Of the current drinkers, the majority (69.1%) were at either moderate or high health risk from alcohol use.* Conclusion*. Alcohol consumption is prevalent in semirural communities in Nigeria and the majority of these drinkers are at moderate or high health risk. Screening, brief intervention, and referral for treatment for unhealthy alcohol use should be integrated into community care services in Nigerian rural communities.

## 1. Introduction

Alcohol consumption is a well-known part of the Nigerian culture and frequently part of festivals and celebrations [1] and, within the past decade, there are indications that there has been a rapid increase in alcohol production and importation as well as its consumption across all age groups [2]. For the year after 1995, the unrecorded alcohol consumption was estimated to be 3.5 litres pure alcohol per capita for population older than 15 in Nigeria [2]. Alcoholic consumption continues to experience strong growth in Nigeria as a result of the aggressive marketing activities of leading players, and drinking is widely considered a part of social activities; therefore, most consumers drink unaccompanied [2]. The entry of several new local brews whose alcohol concentration and ingredients are not yet clearly identified has emerged as another public health issue [3]. Nigeria's population of about 160 million continues to grow at an annual rate of 3% [4]. This creates an ever increasing drinking population. Similar to the Western World, alcohol is also a factor in a large proportion of injuries and road accidents in Nigeria [5] and is also associated with physical health problems [1]. Prospective studies among European and North American populations show that alcohol consumption, especially heavy drinking, is associated with pancreatitis, liver cirrhosis, tuberculosis, pneumonia, injuries, malignancies, and psychiatric morbidity [6, 7]. Therefore, the estimation of prevalence and correlates of alcohol consumption is a systematic prerequisite step in the direction of planning an effective intervention program for the target drinking population. However, there is limited large-scale evidence on prevalence and the correlates of alcohol use in Nigeria, particularly in rural provinces where majority of Nigerians live. While a review of different local studies conducted in primary health care settings in urban areas highlighted high prevalence of alcohol use and associated factors such as male gender, increasing age, low educational level, and marital status [1, 8, 9], there is a need to examine the magnitude of alcohol use and correlates in settings where most Nigerians live in order to influence the public health of Nigerians.

We report here the prevalence and correlates of alcohol consumption and investigate the relationship between alcohol consumption and sociodemographic and associated level of health risks among 1,203 men and women, using cross-sectional data collected during the baseline survey of the “Effectiveness of ASSIST Linked Brief Intervention on Harmful and Hazardous Alcohol Use in Two South-West Rural Communities in Nigeria: A Non-Randomized Intervention Study.”

## 2. Methods

### 2.1. Study Area

The study site was in Ibadan, Oyo State. Ibadan is the capital of Oyo State, Nigeria, and it is the third largest city in Nigeria.

### 2.2. Study Design

This was a follow-up, nonrandomized intervention study. However, we report, herein, baseline measures of hazardous and harmful use with their sociodemographic correlates.

### 2.3. Principles for Recruitment

#### 2.3.1. Inclusion/Exclusion Criteria

The inclusion criteria for the study were both male and female alcohol users of age ≥ 15 years and permanent residents of study areas. The exclusion criteria were nonusers of alcohol of age less than 15 years, not willing to get alcohol intervention, and not a permanent resident of the study areas.

#### 2.3.2. Procedure

We obtained ethical approval for the study from the Ethics and Research Committee of the Ministry of Health, Oyo State of Nigeria; accent was obtained for participants between 15 and 18 years and consent from those over 18 years of age. Using systematic stratified sampling method, two semirural local governments in Ibadan were selected between October 2010 and April 2011. The selected semirural local governments were Lagelu local government (local government A) and Akinyele local government (local government B) areas of Ibadan. The classification was according to National Population Commission in Nigeria, based on population and fund allocation.

All the 11 LGA were classified into urban or semirural each during the first stage of the study. There are five urban local governments and six semirural local government areas in Ibadan. In the second stage, one local government was randomly chosen from the six semirural local government areas. In the third stage, four enumeration areas were systematically selected as clusters. The fourth stage involved the mapping and numbering of all buildings in each of the selected enumeration areas. All households within each building were serially listed in the form specifically designed for the purpose. After getting the list of the households, simple random sampling was used to identify the households that fell within the sample. Regular households were distinguished from institutional households. All eligible respondents, who were 15 years and above in each household, were selected and were interviewed by CHEW using the questionnaires including ASSIST after they gave permission/consent. After administration of the sociodemographic questionnaire, participants were also screened with Alcohol, Smoking and Substance Involvement Screening Test (ASSIST).

### 2.4. Alcohol, Smoking and Substance Involvement Screening Test (ASSIST)

The ASSIST was used to determine the prevalence of alcohol consumption and associated level of harm. The ASSIST was developed by the World Health Organization for alcohol and drug screening in high prevalence settings [8]. According to the ASSIST manual, a score of 0–10 for drugs is regarded as lower risk, 11–26 as moderate risk, and 27+ as high risk. However, in this study, the level of harm was generated using a mean ASSIST score at all the phases of the study. For the purpose of this study, current alcohol use was regarded as use in preceding 30 days.

### 2.5. Measures

#### 2.5.1. Sociodemographic Data

We used a set of precoded and pretested sociodemographic questionnaires to elicit sociodemographic characteristics from the respondents such as age, marital status, socioeconomic class, and years of education.

#### 2.5.2. Prevalence of Alcohol Use

Data on both lifetime and current alcohol consumption were obtained using the ASSIST [8].

### 2.6. Data Analysis

For our univariate analysis, the association between sociodemographic variables and both lifetime and current alcohol use was determined using Pearson's chi square statistics.

Multivariate analysis was carried out using binary logistic regression analysis, using variables that were significant during univariate analysis to determine association with current alcohol use. All analyses were by SPSS version 13.0 [9].

## 3. Results

A total of 1329 youths and adults were selected and invited to participate in this study. Out of these, 1213 completed the questionnaires, giving a response rate of 91.3%. At baseline, responses were incomplete in ten questionnaires and so final analysis was carried out on 1203 questionnaires at baseline.

The mean age of respondents at baseline was 24.45 ± 9.23 years, 623 (51.8%) were males, 796 (66.2%) were married, 570 (47.4%) had at least some secondary education, 513 (42.6%) were from low socioeconomic country, 598 (49.7%) from low average socioeconomic group, 400 (33.3%) were unemployed, and 516 (42.9%) were Christians (Table 1).

Prevalence of lifetime alcohol use was 697 (57.9%), lifetime alcohol use was more prevalent in men *p* < 0.001, married respondents *p* = 0.003, respondents with low level of education *p* < 0.001, respondents of low socioeconomic status, those who were unemployed *p* = 0.03, and the Christians *p* = 0.001 (Table 1).

Prevalence of current alcohol use was 285 (23.7%); current alcohol use increased with increasing age, *p* = 0.02, was commoner in males, *p* = 0.003, the unmarried, *p* < 0.01, those with formal education, *p* = 0.003, those from low socioeconomic group, *p* = 0.01, the unemployed *p* < 0.001, and the Christians *p* < 0.01 (Table 2).

**Figure 1:**
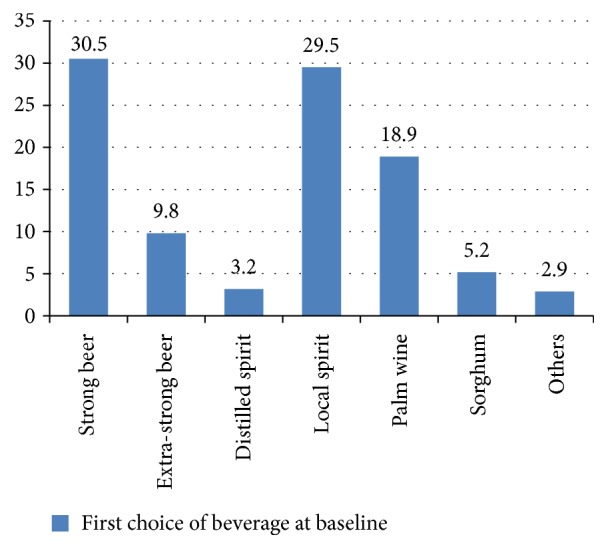
First choice of beverage at baseline.

Of current users, strong beer was first choice of beverage in 30.5% of them and other beverages predominantly local cocktails were the least (2.9%) (Figure 1).

Prevalence of current alcohol use was 285 (23.7%), 88 (30.9%) were at low health risk, 161 (56.5%) were at moderate health risk, and 36 (12.6%) were at high health risk (Table 3).

Multivariate analysis revealed that older age group, female gender, high average socioeconomic group, and high socioeconomic group were protective factors, while being unmarried was a risk factor.

At 3 months, older age group, female gender, high average socioeconomic group, and high socioeconomic group were protective factors, while being unmarried was risk factor (Table 4).

## 4. Discussion

This is the report of baseline measures of a single arm nonrandomized intervention study that aimed to determine in semirural community settings the prevalence and correlates of alcohol use as well as the effectiveness of ASSIST Linked SBIRT on harmful and hazardous alcohol use in the rural youth and adult dwellers and most probably the first in Sub-Saharan Africa.

We found that the prevalence of lifetime alcohol use was 57.9% and current alcohol use 27.3% among the participants. Our finding was in agreement with the lifetime prevalence of alcohol (56%) by Gureje and colleagues [10], in a multicentre study across all cultures in Nigeria. However, our reported current alcohol use is almost twice that reported (14%) by Gureje et al. [9]. This could be a reflection of the difference in the instruments used or in spread of centres used. It could also be a reflection of increase in alcohol consumption over time. Our self-reported lifetime and current uses are lower than lifetime alcohol use (66.6%) and current use (62.2%) from a study in Togo, a neighbouring country [11]. This could be because the Permanent Mandates Commission of the League of Nations could not effectively check France and the European companies operating in Togo, from importing liquor to the country because of the recognized economic gains from liquor trade for the economy of France and other European countries [12].

We found that current drinking was associated with younger age, male gender, being unmarried, low educational status, low or low average socioeconomic class, Christianity, and unemployment. Our findings are in line with those of Gureje et al. [9], studies conducted in primary care settings in Nigeria [13], as well as in community [10]. These findings are also consistent with alcohol surveys conducted in Togo, a neighbouring country [11], as well as previous studies in Nigeria [14]. In consonance with our study, Grittner and colleagues [15] opined that people with lower socioeconomic status (SES) could be more vulnerable to problems related to alcohol consumption.

The most common consumed alcoholic beverage is beer and local spirits and this finding contrasts with the 1998 study in Ibadan by Bennett and colleagues [14] that found palm wine and beer. However, the use of local spirits in this study is in consonance with findings from previous Nigerian surveys [1]. Factors that mediate these observed patterns of beverage choice may include cost of distilled spirits and advertisement by local brewers.

We also found that more than two-thirds of the current drinking population were moderate or high risk drinkers. This is pertinent considering that drinking alcohol is associated with a risk of adverse health consequences such as alcohol dependence, cancers, and injuries [16, 17] and, in semirural or rural settings, there is limited access to health care. Although some primary care attendees may seek consultation for some of the physical health consequences of hazardous drinking, clinicians who are less likely to screen them for alcohol use talk less of initiating an intervention for their alcohol misuse [18].

The multivariate analyses conducted in this study highlight protective and risk factors for alcohol use among dwellers in two semirural communities and provide important epidemiological data in terms of identification of a population who require special attention in terms of substance use prevention and intervention programs.

Our findings have policy implications. Policies that address poverty and improve socioeconomic indices such as increase in job creation and employment and broadening of social network are likely to impact on the level of alcohol use among dwellers in the rural settings in addition to those in the urban settings. The integration of these policies with evidence based alcohol policies that encapsulate demand control, supply control, and harm reduction will go a long way to reduce risky alcohol use and the adverse consequences of alcohol consumption. We are, however, cautious that a cross-sectional study cannot underscore a clear causative inference.

Furthermore, there is the need for multiround cross-sectional studies to evaluate whether the prevalence of drinkers is increasing and whether those who drink are increasing their consumption intensity. An understanding of these trends will guide health promotion and preventive strategies and further lend support for programmes for screening and brief intervention and more intensive programmatic interventions.

Our study was limited by a number of factors. One, we did not allocate any diagnosis to the alcohol users; therefore it was difficult to validate the level of health risk with the diagnosis of alcohol use disorder. Also the external validity of the prevalence rates is limited because of diverse sociocultural characteristics of the Nigerian population.

In conclusion, the prevalence of unhealthy alcohol use is high in a representative sample of semirural communities and correlates include male gender, being unmarried, low educational status, low or low average socioeconomic class, Christianity, and unemployment. This should inform policy decisions to address the magnitude of problematic alcohol use in rural settings, where majority of youth and adult in Nigeria live.

## Figures and Tables

**Table 1:** Sociodemographic characteristics of lifetime drinkers.

Variables	*N*	Lifetime use (*n* = 697)	%	*χ* ^2^	Sig.
Age					
<25	508	295	58.1	0.1	0.9
25–34	256	153	59.8		
35–44	158	90	57.0		
45–54	120	70	58.3		
55–64	111	66	59.5		
>64	50	28	56.0		
Gender					
Male	623	394	63.2	14.7	<0.001
Female	580	303	52.2		
Marital status					
Married	796	437	54.9	8.5	0.003
Not married	407	260	63.9		
Setting					
Local government A	487	255	52.3	0.5	0.5
Local government B	716	392	54.8		
Education					
0	119	100	84.0	29.0	<0.001
1–6	431	277	62.4		
7–12	570	288	50.5		
>12	83	32	38.6		
Socioeconomic group					
Low	513	320	62.4	15.7	<0.001
Low average	598	339	56.7		
High average	63	28	44.4		
High	29	10	34.5		
Employment					
In employment	803	447	55.7	4.8	0.03
Unemployed	400	250	62.5		
Religion					
Christianity	516	326	27.5	9.8	0.001
Islam	687	371	20.8		

**Table 2:** Sociodemographic characteristics of current drinkers.

Variables	*N*	Current use (*n* = 285)	%	*χ* ^2^	Sig.
Age					
<25	508	133	26.2	13.5	0.02
25–34	256	61	23.8		
35–44	158	36	22.8		
45–54	120	21	17.5		
55–64	111	15	13.5		
>64	50	6	12.0		
Gender					
Male	623	170	27.3	8.8	0.003
Female	580	115	19.8		
Marital status					
Married	796	170	21.4	6.7	<0.01
Not married	407	115	28.3		
Setting					
Local government A	487	78	16.0	0.2	0.7
Local government B	716	107	14.9		
Education					
0	119	44	37.0	13.0	0.003
1–6	431	101	23.4		
7–12	570	120	21.1		
>12	83	20	24.1		
Socioeconomic group					
Low	513	146	28.5	11.4	0.01
Low average	598	122	20.4		
High average	63	12	19.4		
High	29	5	17.2		
Employment					
In employment	803	165	20.5	12.6	<0.001
Unemployed	400	120	30.0		
Religion					
Christianity	516	142	27.5	6.9	<0.01
Islam	687	143	20.8		

**Table 3:** Current alcohol use and level of health risk.

Variables	*N* = 1203	Mean (SD)
A: score 0–10 on ASSIST^L%^	88 (30.9)	20.52 (5.42)
B: score 11–26 on ASSIST^M%^	161 (56.5)	38.38 (6.06)
C: score 27+ on ASSIST^H%^	36 (12.6)	27.21 (7.21)

L: low risk of health problems, M: moderate risk of health problems, and H: high risk of health problems.

**Table 4:** Odd ratio for current alcohol use.

Variation	OR	CI	Sig.
Age			
<25	1		
25–34	0.58	0.33–1.00	0.05
35–44	0.59	0.22–0.94	<0.05
45–54	0.41	0.11–0.88	0.04
55–64	0.35	0.09–0.87	0.04
>64	0.21	0.08–0.75	0.02
Gender			
Male	1		
Female	0.32	0.008–0.62	0.01
Marital status			
Married	1		
Not married	3.32	1.66–6.12	0.01
Socioeconomic group			
Low	1		
Low average	0.89	0.29–1.44	0.16
High average	0.32	0.09–0.87	0.03
High	0.24	0.08–0.72	0.02

## Additional Points

What is known is that the problem of alcohol use is a huge public health issue worldwide and among Nigerian adults. What the study adds is that prevalence of alcohol consumption in Nigeria is guided by demographic variability.
